# DNA-Based Identification of Eurasian *Vicia* Species Using Chloroplast and Nuclear DNA Barcodes

**DOI:** 10.3390/plants11070947

**Published:** 2022-03-31

**Authors:** Irene Bosmali, Georgios Lagiotis, Nadia Haider, Maslin Osathanunkul, Costas Biliaderis, Panagiotis Madesis

**Affiliations:** 1 Centre for Research and Technology Hellas, Institute of Applied Biosciences, 57001 Thessaloniki, Greece; eirinimposmali@certh.gr (I.B.); glagiotis@certh.gr (G.L.); 2 Laboratory of Food Chemistry and Biochemistry, Department of Food Science and Technology, School of Agriculture, Aristotle University of Thessaloniki, 54124 Thessaloniki, Greece; biliader@agro.auth.gr; 3 The Department of Molecular Biology and Biotechnology, Atomic Energy Commission (AEC), Damascus 6091, Syria; ascientific1@aec.org.sy; 4 Department of Biology, Faculty of Science, Chiang Mai University, Chiang Mai 50200, Thailand; maslin.cmu@gmail.com; 5 Research Center in Bioresources for Agriculture, Industry and Medicine, Chiang Mai University, Chiang Mai 50200, Thailand; 6 Laboratory of Molecular Biology of Plants, School of Agricultural Sciences, University of Thessaly, 38446 Volos, Greece

**Keywords:** DNA barcoding, ITS2, *rpoC1*, *trnL*, *Vicia*

## Abstract

Many legume species of the *Vicia* L. genus (Fabaceae Lindl.) are key components of the Mediterranean diet and have an integral role in sustainable agriculture. Given the importance of the *Vicia* species for Eurasian culture, it is necessary to implement methodologies, such as DNA barcoding, that can enable the effective authentication and identification of species in the genus. In this study, we analysed the chloroplast *trnL* and *rpoC1*, as well as the nuclear ITS2 DNA barcoding regions, to identify 71 *Vicia* specimens of Eurasian descent. Both the *trnL* and ITS2 regions were highly effective in discriminating the analysed taxa, while the more conserved *rpoC1* region could not identify all of the selected species due to high sequence conservation or non-annotated or absent *rpoC1* species sequences in GenBank. A dendrographic representation of the generated *trnL* data showed sufficient clustering for most of the analysed taxa, although some topological discrepancies were observed. ITS2 and *rpoC1* reconstructions were also used for resolving the topological discrepancies observed in the *trnL* tree. Our analysis suggests that a combination of DNA barcoding regions is essential for accurate species discrimination within the *Vicia* genus, while single-locus analyses do not provide the necessary resolution.

## 1. Introduction

Legumes are the second most important crop after cereals in terms of agricultural production, human nutrition, and animal feed [1]. Many leguminous species have been an integral part of the human diet since ancient times due to their low fat and high protein content, richness in fibre and other nutrients, as well as their anticarcinogenic and prebiotic potential [1,2,3,4,5]. Furthermore, legumes are a nutrient-rich feed for domesticated animals. Apart from the use of legumes for human or animal consumption, many species are also utilized in agricultural practices, such as crop rotation, given many species form symbiotic relationships with nitrogen-fixing bacteria, thus replenishing soil nitrogen levels. Finally, legumes are also an invaluable source of nutraceuticals and pharmaceuticals [4,6,7], and are important in the production of other industrial products [8]. Legume seeds can also be fractionated into high protein-, fibre-, and starch-based raw ingredients for the food, pet, and paper-making industries due to their physicochemical properties and nutritional characteristics [9,10,11].

Leguminous plants belong to the Fabaceae (Leguminosae Jussieu) family, which is one of the largest plant families, including more than 10% of the known eudicots [12]. The family includes 670 genera and approximately 20,000 species [13]. Fabaceae has been traditionally divided into three subfamilies (Papilionoideae L., Mimosoideae DC., and Caesalpinioideae DC.). Legumes of the tribe Fabeae Rchb. of the subfamily Papilionoideae are distributed worldwide throughout Eurasia, Africa, and America. The tribe Fabeae is composed of the *Vicia* L., *Lathyrus* L., *Lens* Mill., *Pisum* L., and *Vavilovia* Al. Fed. genera [13], and likely originated in the eastern Mediterranean region during the Miocene, dispersing several times into Eurasia, and a few times to the American and African continents [14,15].

Amongst the various Fabeae genera with wide distribution in the Mediterranean region, *Vicia* shows high levels of species diversity [16,17]. Although the number of total species has not yet been conclusively defined [18], it is estimated to be approximately 200 species. The *Vicia* species show a widespread distribution in the temperate zones of the northern hemisphere and the tropical regions of South America [18]. Although the genus does not largely include any threatened species, there are many rare species restricted to particular habitats [19]. Given that human activity has degraded many of the major centres of *Vicia* species diversity, local diversity conservation is important in preserving the genetic variation of the genus for breeding purposes, especially for wild species. For example, several wild *Vicia* species in Syria were threatened with local extinction, and more recent surveys show that other species are also under threat [20]. With the scope of preserving the invaluable species diversity of the genus, especially for endemic species, it is important to correctly and effectively discriminate the closely related *Vicia* species.

Several morphological and DNA-based descriptors (e.g., consensus chloroplast simple sequence repeats (ccSSRs) [21]) of diversity have been used to discriminate amongst species in the *Vicia* genus. Morphological characteristics, such as flower traits, pollen morphology, leaf structure, and legume shape, have been considered to be important taxonomic traits for correct species identification in the genus [17,22,23,24,25]. Nevertheless, phenotypic identifications require specialized taxonomic expertise and a detailed description of species morphology at various developmental stages. There are many limitations on morphology-based species characterization, and it can be impossible to describe the underlying genetic variation. These limitations become even more pronounced for closely related species with very similar morphologies and species with intermediate phenotypes [26], as well as for recently diverging species or species generated by hybridization [27]. Given the large-scale karyotype structure variation observed amongst several *Vicia* species, karyological and cytological studies have also been carried out for *Vicia* taxonomy [28,29]. However, the varying results from earlier studies in resolving species discrimination necessitates the application of more reliable and effective methods to secure the invaluable Vicia species diversity.

DNA barcoding, which involves the sequencing of small nuclear and chloroplast genome regions (DNA barcodes) amongst diverse samples, results in an easily comparable dataset that enables species discrimination, and it has been proposed to be an effective methodology for the taxonomic identification, description, revision, and reordering of species [30,31]. Although there has been extensive research into the application of single and multi-loci analyses in plant species identification [32,33,34,35,36], there is still no consensus on barcodes that are universally ideal for all plant species, especially in the *Vicia* genus. In several plant species, the variable chloroplast DNA regions, such as *trnL*, *rbcL*, and *matK*, are usually ideal and have been used effectively in species discrimination [33,35,37,38,39]. Apart from the various chloroplast regions, the nuclear ITS region has also been proposed to be an ideal barcode for species discrimination, given that it has high PCR amplification efficiency and is highly variable in many plant species [30]. The ITS region was previously used to effectively discriminate amonst several medicinal plants in the Fabaceae family [40]. Within the *Vicia* genus, several studies have demonstrated the effectiveness of this region in species identification and phylogenetic reconstructions [15,41,42,43]. However, it becomes increasingly apparent that there are some limitations in single loci analyses, and the combination of multiple barcodes may provide higher discrimination accuracies, especially for closely related species [44].

The combination of the chloroplast regions *trnL* and *rpoC1*, as well as the nuclear ITS2 region, has been previously used for the discrimination of major Mediterranean legume species, with *trnL* and ITS2 showing the highest level of sensitivity [39]. More specifically for *Vicia*, the ITS2 and several regions of the chloroplast genome were previously used for the taxonomic classification of several species in the genus [45,46,47]. More recently, the combination of the barcoding regions ITS2, *matK*, and *rbcL* was also evaluated along with morphological taxonomic traits for their efficacy in discriminating among South Korean *Vicia* species [48].

Apart from species discrimination, DNA barcoding data have also been used for inferring and resolving phylogenetic relationships in several genera in the Fabaceae family, including *Vicia*. Phylogenetic analyses based on both morphological descriptors of diversity and DNA data showed that the Eurasian species are likely to have originated in the eastern Mediterranean or the western Asiatic regions and the New World species were derived from lineages that invaded America [1,41]. The divergence of the two groups occurred relatively recently [41] and was likely the result of three independent invasion events [12,49]. Nevertheless, *Vicia* species phylogeny is still inconclusive, owing to the taxonomic complexities that emerge from incongruent phylogenetic analyses and the high intraspecific genetic diversity observed in the genus [1], as well as the classification of several taxa as subspecies and races [50] or aggregates [50,51,52].

To verify the species identity of several *Vicia* specimens of Eurasian descent in local seed banks, we implemented DNA barcoding analyses using the *trnL* and *rpoC1* plastid regions, as well as the nuclear ITS2 region. Species discrimination was carried out in terms of sequence similarity (>90%) with indicative GenBank entries, and Maximum Likelihood (ML) dendrograms were utilized for visualizing the sequence differences amongst the studied specimens. Our analysis indicates that the combination of both sequence similarity assessments and dendrographic representations allows for more accurate species discrimination in the *Vicia* genus.

## 2. Results

To support species identification for 71 indicative Eurasian *Vicia* specimens from local seed banks, DNA barcoding analysis was implemented using, initially, the *trnL* region. The *trnL* DNA barcoding region was successfully amplified and sequenced for all 71 specimens, generating reads of approximately 362 bp in length. A Basic Local Alignment Search Tool (BLAST) analysis of the generated *trnL* sequences against GenBank entries enabled us to confidently identify the *Vicia* specimens at the species level with high-percentage identity values above 90% for most of the specimens (Table 1).

**Figure 1:**
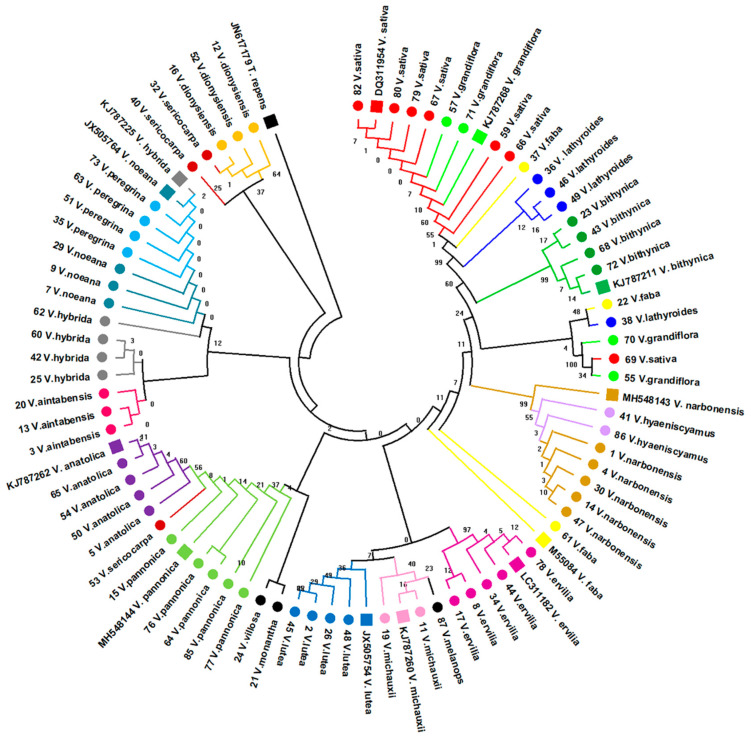
Dendrogram based on the *trnL* alignment matrix generated for 71 specimens across 20 *Vicia* species. The tree was generated using the Maximum Likelihood method and the Tamura-Nei model with +G = 0.693. The tree with the highest log likelihood (−908.72) is shown. Branch labelling represents the percentage of replicate trees in which the corresponding taxa clustered together (1000 bootstrap replicates). The *Trifolium repens trnL* sequence (GenBank acc. number: JN617179) was used as an outgroup to root the tree. The Taxa are colour-coded at the species level for visualization purposes. The *Vicia* species analysed in this work are labelled with circle markers. Numbers in species labelling correspond to sample ID (Table 1). GenBank-derived reference sequences are indicated by square-shaped markers and accession numbers present in the taxon labelling.

To graphically represent the sequence differences amongst the studied specimens, a multiple sequence alignment was assembled for the generated *trnL* sequences of the 71 *Vicia* specimens, along with *trnL* reference sequences from GenBank. The resulting alignment generated a matrix of 492 sites in total, with 207 variable and 130 parsimony-informative sites (Table S1). When studying the generated *trnL* ML tree, most specimens of the same *Vicia* species showed distinct clustering (Figure 1).

However, there were some discrepancies observed in the topology of several species, especially for the *V. faba* samples, which were scattered throughout the tree (Figure 1). Furthermore, individual specimens, such as *V. sericocarpa* (specimen 53) and *V. lathyroides* (specimen 38), did not cluster with other specimens of the same species (Figure 1). Despite the apparent clustering of specimens in the clade formed by the *V. aintabensis*, *V. hybrida*, *V. noeana*, and *V. peregrina* species, there were very few differences in the *trnL* sequence in these species that could allow further sub-clustering; hence, bootstrap support is very low in this clade (Figure 1). Similar cases for topology were also observed for the *V. sativa* and *V. grandiflora* species (Figure 1). Despite some taxon misplacements, broader patterns can be clearly observed. According to the *trnL* tree, *V. dionysiensis* shows an early divergence, followed by a clade that radiates to species with very similar *trnL* sequences (*V. hybrida*, *V. aintabensis*, *V. noeana*, and *V. peregrina*), a clade that splits to the *V. anatolica* and *V. pannonica* species, a clade that primarily contains the *V. lutea*, *V. michauxii*, and *V. ervilia*, and finally, a clade that diverges into the *V. narbonensis*, *V. bithynica, V. lathyroides*, and *V. sativa/V. grandiflora* species (Figure 1).

**Figure 2:**
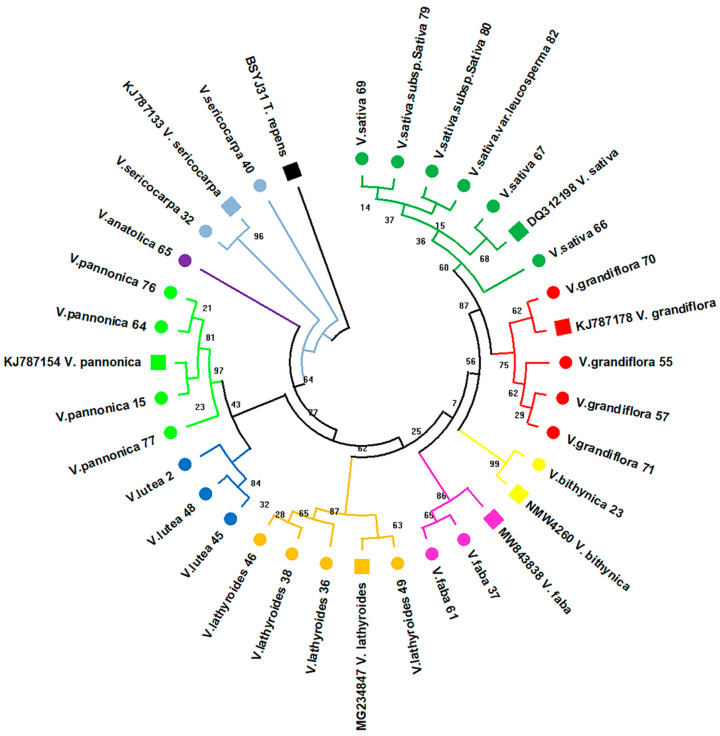
Dendrogram based on the ITS2 alignment matrix generated for 27 specimens across 9 *Vicia* species. The tree was generated based on the Maximum Likelihood method and the Kimura 2-parameter model with +G = 2.119. The tree with the highest log likelihood (−1112.59) is shown. Branch labelling represents the percentage of replicate trees in which the corresponding taxa clustered together (1000 bootstrap replicates). The *Trifolium repens* ITS2 sequence (GenBank acc. number: BSYJ31) was used as an outgroup to root the tree. Newly generated *Vicia* species sequences are labelled with circular markers. Number annotations in species labelling correspond to sample IDs (Table 2). Reference sequences from GenBank with the corresponding accession numbers are marked with square-shaped markers.

To further verify broad tree topologies and resolve taxon discrepancies, the ITS2 and partial *rpoC1* regions were also analysed for selected taxa. For these analyses, specimens were primarily selected for their misplacement on the *trnL* tree, especially those that did not form any apparent clustering, such as *V. faba* (Figure 1). Individual specimens with an unsuccessful amplification of the ITS2 and *rpoC1* regions were excluded from the subsequent analyses, although alternative representatives of the species were used in this case. A BLAST analysis of the generated ITS2 sequences for the selected taxa showed high levels of similarity with the corresponding reference sequences from GenBank (Table 2). The alignment of those sequences with reference sequences generated a matrix of 436 bp, with 200 variable and 37 parsimony-informative sites (Table S1). In contrast to *trnL*, the ITS2 tree resolved the topological discrepancies observed in the *V. faba* and *V. lathyroides* clades, with all the specimens of the same species displaying distinct clustering (Figure 2). Despite the lack of strict clustering for the *V. sericocarpa* species, the ITS2 sequences in these specimens are still more similar with each other than those from the other specimens; hence, the *V. sericocarpa* branches did not interject the well-defined clusters on the ITS2 tree, in contrast to what has been observed in the *trnL* tree (Figure 1 and Figure 2).

Overall, in the context of the studied species, the overall ITS2 tree topology retains the broad pattern observed in the *trnL* tree, with an earlier divergence of the *V. sericorpa* species followed by *V. anatolica,* a clade diverging to *V. lutea* and *V. pannonica*, as well as a clade comprised of the rest of the species (Figure 2). It is noteworthy that the divergence of *V. faba*, according to the ITS2 dendrogram, is placed before that of *V. bithynica*, as well as those of *V. sativa* and *V. grandiflora* clades (Figure 2).

To further resolve the taxon discrepancies observed in the *trnL* tree with a more conserved region, BLAST analysis and dendrographic reconstruction were also performed using part of the *rpoC1* region, which encodes for the beta subunit of the DNA-dependent RNA polymerase. Despite the successful amplification and sequencing of the *rpoC1* region for all of the *Vicia* specimens examined (Table 3), there were no available data in the GenBank repository for most of the tested species. As such, verification of species identity by sequence similarity using this region was not feasible. Nevertheless, the two species for which data exist, namely *V. faba* and *V. sativa*, showed 100% similarity with high bit score values, as expected for a conserved DNA barcoding region (Table 3).

**Figure 3:**
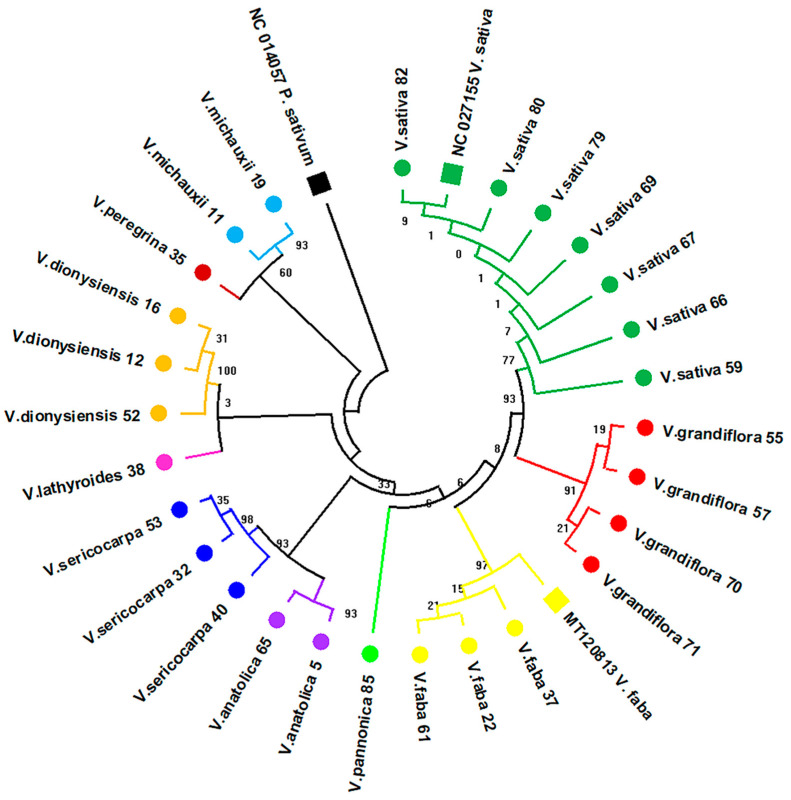
Dendrogram based on the *rpoC1* alignment matrix generated for 27 specimens across 10 *Vicia* species. The tree was generated using the Maximum Likelihood method and the Jukes–Cantor model with +G = 0.05. The tree with the highest log likelihood (−927.88) is shown. Branch labelling represents the percentage of replicate trees in which the corresponding taxa clustered together (1000 bootstrap replicates). The *Pisum sativum rpoC1* sequence (GenBank acc. number: NC014057) was used as an outgroup to root the tree. The *Vicia* species sequences generated in this work are labelled with circular markers. Number annotations in species labelling correspond to sample IDs (Table 3). Reference sequences from GenBank with the corresponding accession numbers are labelled with square-shaped markers.

The alignment of the corresponding sequences with reference species sequences from GenBank generated an alignment matrix of 442 bp with 25 parsimony-informative and 37 variable sites (Table S1), which indicates very low variation amongst the analysed sequences, as expected for a rather conserved region. On the basis of the generated *rpoC1* dendrogram, most of the recently diverged species, such as *V. sativa, V. grandiflora*, and *V. faba*, were clearly separated into distinct clades, showing topological agreement with the ITS2 tree (Figure 2 and Figure 3).

However, the *V. lathyroides*, *V. peregrina*, and *V. michauxii* species showed much earlier divergence than that observed in the *trnL* and ITS2 trees (Figure 1 and Figure 2). Especially for *V. michauxii* and *V. peregrina*, the *rpoC1* tree shows an even earlier divergence than that of the *V. dionysiensis* clade (Figure 3), which was shown to have the earliest divergence amongst the analysed *Vicia* species in the *trnL* tree (Figure 1).

## 3. Discussion

Legume crops and their products are important elements of the human diet, an essential animal feed, as well as an integral component of sustainable agriculture. Many leguminous species of the *Vicia* genus have been domesticated and are commonly used in various Protected Designation of Origin (PDO) and Protected Geographical Indication (PGI) products [53], which necessitates the application of methodologies that will enable the effective authentication and identification of the product’s composition. Furthermore, a comparative analysis of morphological, biochemical, karyotypic, and genetic characteristics of several *Vicia* species revealed inconsistencies in the resulting classifications within the genus [54,55,56,57,58], which indicates that more reliable methodologies using genetic markers with general taxon specificity are required for species identification.

DNA barcoding analysis was proven to be one of those methods capable of discriminating among species on the basis of the sequence of small DNA region(s) [30,37,38]. This methodology was successfully used not only for species identification and forensic analyses, but also for biodiversity and phylogenetic studies [59,60]. DNA barcoding in plants most commonly uses chloroplast sequences, which are species-specific [37,38]. Unlike the nuclear genome that follows Mendelian inheritance, chloroplasts do not undergo meiotic recombination and show maternal inheritance in the majority of the angiosperms [61]. Furthermore, the chloroplast genome is sufficiently rich in polymorphic sequences, which facilitates the conduct of evolution studies and species discrimination in plants [62,63,64]. Nevertheless, given the lack of a single universal barcode that can be effectively used for all plant species [65], the application of multi-loci approaches, utilizing both nuclear and chloroplast DNA barcodes, has significantly increased the identification accuracies, especially for closely related species [44].

To verify the accuracy of species identification for indicative Eurasian *Vicia* specimens deposited in local seed banks, we implemented DNA barcoding analyses using the highly variable *trnL* and ITS2 DNA barcoding regions. Both DNA barcodes have rich representation in the GenBank repository for many plant species, including species in the *Vicia* genus, thus allowing for a more accurate species discrimination using sequence similarity (>90%). The rather conserved chloroplastic *rpoC1* region was also used, although it is not the most optimal barcode for species discrimination in the Fabaceae [39], given that it can facilitate a more optimal interspecific alignment and tree topology than the highly variable *trnL* and ITS2 regions.

More specifically, to facilitate species identification for the 71 Eurasian *Vicia* specimens studied in this work and verify their morphological identification prior to seed bank submission, we initially analysed the chloroplast *trnL* DNA barcoding region in these specimens. The *trnL* region was chosen as the primary barcode of our analysis, given that it has the desirable characteristics for DNA barcoding, especially for sample preparations with compromised DNA quality [32,35], and was also used successfully for species discrimination in the Fabaceae family, including *Vicia* [15,39]. Using the BLAST analysis of the generated *trnL* sequences, the majority of the specimens were successfully discriminated at the species level with notably high similarity values, which were higher than 90% identity (Table 1), further indicating that the *trnL* region can be reliably used for species discrimination in the *Vicia* genus. The derived *trnL* tree showed an overall agreement in the topology of the taxa with the corresponding reference entries, although some discrepancies were noted for several taxonomic groups (Figure 1). Although the generated dendrogram is not a phylogenetic tree per se, it can still be used to facilitate species discrimination, especially for inconclusive BLAST results. As such, pertaining to the *trnL* dendrogram, the most pronounced incongruences were observed for the *V. sativa* and *V. grandiflora* clades that are not clearly defined, as well as the *V. faba* and *V. sericocarpa* species, which do not form distinct clusters, with several individual specimens being placed throughout the tree (Figure 1). Very recently, Han et al. (2021) inferred similar topologies when testing the phylogeny of species in the *Vicia* genus, with *V. sativa* and *V. faba* showing the most recent divergence and the clades radiating to *V. panonica*, *V. aintabensis*, and *V. peregrina* having an earlier divergence [48].

In an attempt to further resolve the topological incongruences observed in the *trnL* tree, especially for the *V. faba*, *V. grandiflora*, *V. lathyroides*, *V. sativa*, and *V. sericocarpa* species, and to further support species discrimination amongst the specimens, the ITS2 region was also analysed for the selected taxa. The selection of taxa for the ITS2 analysis was primarily based on specimens that did not form any apparent clustering and were spread across the *trnL* tree, such as *V. faba* and *V. sericocarpa* (Figure 1). Several other taxa were also selected from various taxonomic groups, despite their placement on the *trnL* tree, in order to preserve the overall dendrographic topology, and broad topological comparisons can be made between the two barcoding regions. Kress et al. (2005) have reported the use of the nuclear ITS2 region as a suitable barcode for flowering plants, although it has been problematic for some species [37]. Furthermore, the ITS2 region was regarded as successful for species discrimination in the Fabaceae family [39,43] and more specifically in the *Vicia* genus [41,46,48,66]. BLAST analysis using the ITS2 region was also highly effective in discriminating the analysed taxa (Table 2), with remarkably high-percentage identity values (>98%) between the analysed taxa and GenBank reference sequences. This is consistent with previous findings that show both *trnL* and ITS2 as being equally effective in species discrimination for species in the Fabaceae family [39]. The overall topological discrepancies observed in the *trnL* tree have been resolved with high bootstrap support using the ITS2 region (Figure 2). More specifically, the *V. sativa* and *V. grandiflora* clades were sufficiently resolved in distinct clusters (Figure 2). According to the ITS2 tree, the *V. faba* samples showed a more defined clustering, which positions this species divergence in between *V. bythinica* and *V. lathyroides* (Figure 2). This placement of *V. faba* is consistent with previous phylogenetic studies [41,48,66,67,68,69], which further supports the accuracy of the ITS2 tree topology.

Although the ITS2 analysis greatly resolved the general topological incongruences observed in the *trnL* tree, despite being a highly variable region, the conserved *rpoC1* region was also used for selected species, notwithstanding the reported low discrimination efficiency [32]. We argue that a more conserved region of the chloroplast genome would be more effective in discriminating species in the genus. However, in contrast with the other barcoding regions, *rpoC1* could not identify the selected species apart from the *V. sativa* and *V. faba* species, which showed 100% identity (Table 3). This was likely the result of non-annotated or absent *rpoC1* sequences in the GenBank repository. The generated *rpoC1* tree showed consistency with the ITS2 tree pertaining to the topology of the recently diverging *V. sativa*, *V. grandiflora*, and *V. faba* taxa, whilst early diverging clades, such as *V. lathyroides*, *V. peregrina*, and *V. michauxii*, were not congruent between the two barcodes (Figure 2 and Figure 3). Although all of the taxa show high intraspecific bootstrap support and clear intraspecies clustering, the majority of the analysed clades, except for *V. sativa*, *V. grandiflora*, and *V. faba*, show topological discrepancies compared to the rest of the trees present in this study (Figure 3). This finding may be attributed to the fact that *rpoC1* is a highly conserved region to allow for accurate species discrimination for at least some species in the *Vicia* genus. Moreover, the *rpoC1* region was shown to be the least discriminatory when used for species identification amongst several coding and non-coding barcodes within the Fabaceae family [39,40]. Hence, species discrimination within the *Vicia* genus would be difficult to achieve using only this barcode and will require more thorough investigation with more specimens in these groups.

## 4. Materials and Methods

### 4.1. Plant Materials and DNA Extraction

Seeds of 71 specimens corresponding to 20 different species of the genus *Vicia* (Fabeae, Fabaceae) were provided by the Genetic Resources Unit at the International Center for Agricultural Research in the Dry Areas (ICARDA) in Aleppo, Syria, and by the General Commission for Scientific Agricultural Research (GCSAR) in Damascus, Syria. Species selection was based on the availability of *Vicia* species germplasms in the corresponding seed banks. Information on species names and sources of origin, which were derived from the seed bank records, is presented in Table 4.

A total of 5 seeds from each specimen were sown in a 0.5 L plastic pot containing turf (Euroveen B. V., Charleston, SC, USA). Seeds were allowed to germinate under laboratory conditions (21 °C) and grow for 1 month. DNA was extracted from 0.5 g leaf tissue of each specimen using the CTAB method according to Doyle and Doyle (1987) [70]. The DNA concentration was measured with standard spectrophotometric procedures using the Eppendorf BioPhotometer (Eppendorf, Hamburg, Germany). DNA quality and quantity were further assessed with gel electrophoresis on a 1% agarose gel. Samples were then diluted to 20 ng/µL working stocks.

### 4.2. PCR Amplification and Sequence Analysis

PCR amplification was performed to a final volume of 50 μL in a Veritti research thermocycler (Applied Biosystems, Waltham, MA, USA). The reaction mixture contained 20 ng genomic DNA, 1X PCR buffer, 2.5 mM MgCl_2_, 0.2 mM dNTPs mix, 0.4 μM forward and reverse primers, and 1 U Kapa Taq DNA Polymerase (Kapa Biosystems, Wilmington, MA, USA). The primer sequences used for the DNA barcoding analyses are shown in Table S2. PCR cycling was carried out with an initial denaturation step at 94 °C for 3 min, followed by 35 cycles at 94 °C for 30s, 55 °C for 40s, and 72 °C for 1 min, with a final extension step at 72 °C for 2 min. The PCR products were sequenced in two directions with the Big Dye terminator v3.1 Cycle sequencing kit (PE Applied Biosystems, Foster City, CA, USA) in an automated ABI 3730 sequencer (PE Applied Biosystems). Sequence ambiguities were manually corrected using the CHROMAS software version 2.6.6 (Technelysium Pty Ltd., South Brisbane, Australia).

### 4.3. Basic Local Alignment Search Tool (BLAST) Analysis

Species identification, using the sequence similarity approach, was performed using the GenBank database (https://www.ncbi.nlm.nih.gov/genbank/, accessed on 13 May 2021). The default search parameters were used, optimizing for highly similar sequences (megablast). Species identification was regarded as successful when both the species label of the specimen deposited in the local seed banks and the molecular identity according to sequence similarity agreed by >90%. The generated DNA barcoding sequences were submitted to GenBank with accession numbers MZ334891-MZ334961 for *trnL*, accession numbers MZ338297-MZ338323 for ITS2, and accession numbers MZ285764-MZ285790 for *rpoC1.*

### 4.4. Multiple Sequence Alignment and Dendrographic Representation

Multiple sequence alignments were generated for the *trnL*, ITS2, and *rpoC1* sequences of the samples used herein, along with representative reference sequences from GenBank for representative taxa. The Molecular Evolutionary Genetics Analysis X (MEGA X) software version 10.05 [71] with the MUSCLE algorithm was used for alignment generation. A Maximum Likelihood (ML) fitness assessment for 24 substitution models was carried out for each of the alignments using MEGA X (Table S3). The model with the lowest Bayesian Information Criterion (BIC) value for each alignment was selected to subsequently generate the corresponding ML trees with 1000 bootstrap replicates using MEGA X. The Tamura-Nei (T92) model [72] with discrete gamma distributions (+G = 0.693) was used for *trnL*, the Kimura 2-parameter (K2-P) model [73] with discrete gamma distributions (+G = 2.119) was used for ITS2, and the Jukes–Cantor model (JC) model [74] with discrete gamma distributions (+G = 0.050) was used for *rpoC1* tree reconstructions. The *Pisum* and *Trifolium* species in the dendrograms were used as rooting tree outgroups.

## 5. Conclusions

Legumes, such as faba, peas, and lupins, have the potential to replace animal-based protein sources, such as meat, bone, or fish, as well as provide other important seed fractions, namely, starch and fibre, as raw materials for making a variety of formulated products in the food, feed, and cosmetics industries. Given the importance of several *Vicia* species in the Mediterranean diet and the value of such ingredients in local PDO and PGI products, we implemented DNA barcoding analysis using the *trnL,* ITS2, and *rpoC1* regions to discriminate Eurasian *Vicia* species. Our analysis indicates that both the *trnL* and ITS2 can effectively discriminate *Vicia* species, whilst the use of the *rpoC1* region was not capable of identifying most of the analysed species. The use of multi-loci analyses may be more powerful to provide higher discrimination accuracies, especially for the closely related species that single DNA barcodes fail to resolve. Broad dendrographic representations for the analysed taxa were also more congruent when considering topologies reconstructed from the *trnL* and ITS2 data, in contrast to the topologies obtained from *rpoC1*. We expect the work presented herein to enrich the available barcoding data for species discrimination in the *Vicia* genus, which will contribute significantly to the resolution of the still inconclusive phylogeny of the genus, as well as for agricultural and culinarian purposes, especially for the local Eurasian communities.

## Figures and Tables

**Table 1:** *trnL* BLAST analysis of the 71 *Vicia* specimens.

Species/Subspecies Name	Sample ID	GenBank Accession Number	Bit Score	*E*-Value	% Identity
*V. aintabensis* Boiss.	3	MZ334891	737	0	100%
*V. aintabensis* Boiss.	13	MZ334892	702	0	100%
*V. aintabensis* Boiss.	20	MZ334893	697	0	100%
*V. anatolica* Turrill.	5	MZ334894	713	0	99.24%
*V. anatolica* Turill.	50	MZ334895	845	0	99.57%
*V. anatolica* Turill.	54	MZ334896	704	0	99.48%
*V. anatolica* Turill.	65	MZ334897	630	0	99.43%
*V. bithynica* L.	23	MZ334898	501	1E-137	100%
*V. bithynica* L.	43	MZ334899	488	3E-141	100%
*V. bithynica* L.	68	MZ334900	466	4E-127	100%
*V. bithynica* L.	72	MZ334901	516	4E-142	100%
*V. dionysiensis*	12	MZ334902	ǂ	ǂ	ǂ
*V. dionysiensis*	16	MZ334903	ǂ	ǂ	ǂ
*V. dionysiensis*	52	MZ334904	ǂ	ǂ	ǂ
*V. ervilia* L.	8	MZ334905	736	0	100%
*V. ervilia* L.	17	MZ334906	734	0	100%
*V. ervilia* L.	34	MZ334907	652	0	100%
*V. ervilia L.*	44	MZ334908	693	0	100%
*V. ervilia* L.	78	MZ334909	739	0	100%
*V. faba* L.	37	MZ334911	198	7E-52	90.13%
*V. faba* L.	61	MZ334910	307	2E-84	79.41%
*V. faba* L.	22	MZ334912	412	3E-116	89.05%
*V. grandiflora* Scop.	55	MZ334913	377	3E-107	100%
*V. grandiflora* Scop.	57	MZ334914	433	3E-124	100%
*V. grandiflora* Scop.	71	MZ334916	518	9E-150	100%
*V. grandiflora* Scop.	70	MZ334915	481	2E-138	100%
*V. hyaeniscyamus* Mouterde	41	MZ334917	ǂ	ǂ	ǂ
*V. hyaeniscyamus* Mouterde	86	MZ334918	ǂ	ǂ	ǂ
*V. hybrida* L.	25	MZ334919	808	0	100%
*V. hybrida* L.	42	MZ334920	693	0	98.97%
*V. hybrida* L.	60	MZ334921	800	0	100%
*V. hybrida* L.	62	MZ334922	737	0	98.80%
*V. lathyroides* L.	36	MZ334923	449	4E-122	99.59%
*V. lathyroides* L.	38	MZ334924	178	4E-50	89.36%
*V. lathyroides* L.	46	MZ334925	472	8E-129	99.61%
*V. lathyroides* L.	49	MZ334926	459	6E-125	99.60%
*V. lutea* L.	2	MZ334927	761	0	99.76%
*V. lutea* L.	45	MZ334928	704	0	99.23%
*V. lutea* L.	26	MZ334929	739	0	99.50%
*V. lutea* L.	48	MZ334930	730	0	98.31%
*V. michauxii* Spreng.	11	MZ334931	717	0	99.74%
*V. michauxii* Spreng.	19	MZ334932	612	7E-171	99.70%
*V. monantha* Retz.	21	MZ334933	628	0	100%
*V. narbonensis* L.	1	MZ334934	390	2E-104	99.53%
*V. narbonensis* L.	4	MZ334935	392	5E-105	100%
*V. narbonensis* L.	30	MZ334936	514	1E-141	99.65%
*V. narbonensis* L.	14	MZ334937	377	1E-100	100%
*V. narbonensis* L.	47	MZ334938	444	2E-120	99.59%
*V. noeana* Boiss.	7	MZ334939	767	0	99.76%
*V. noeana* Boiss.	9	MZ334940	760	0	100%
*V. noeana* Boiss.	29	MZ334941	773	0	100%
*V. pannonica* Crantz	85	MZ334945	741	2E-179	95.91%
*V. pannonica* Crantz	64	MZ334942	835	0	100%
*V. pannonica* Crantz	76	MZ334943	758	0	100%
*V. pannonica* Crantz	15	MZ334944	719	0	99.74%
*V. pannonica* Crantz	77	MZ334946	765	0	99.52%
*V. peregrina* L.	35	MZ334947	693	0	99.47%
*V. peregrina* L.	51	MZ334948	846	0	100%
*V. peregrina* L.	63	MZ334949	791	0	99.77%
*V. peregrina* L.	73	MZ334950	785	0	99.77%
*V. sativa* L.	66	MZ334952	455	9E-124	94.24%
*V. sativa* L.	59	MZ334951	483	4E-132	99.25%
*V. sativa* L.	67	MZ334953	488	8E-134	99.63%
*V. sativa* L.	80	MZ334956	503	3E-138	100%
*V. sativa* L.	69	MZ334954	523	4E-144	99.31%
*V. sativa* L.	79	MZ334955	420	3E-113	100%
*V. sativa* L.	82	MZ334957	453	3E-123	99.60%
*V. sericocarpa* Fenzl	32	MZ334958	628	7E-176	96.06%
*V. sericocarpa* Fenzl	40	MZ334959	597	2E-166	97.97%
*V. sericocarpa* Fenzl	53	MZ334960	656	0	99.72%
*V. villosa* Roth	24	MZ334961	667	0	99.46%

ǂ Species sequences missing from the GenBank database.

**Table 2:** ITS2 BLAST analysis of selected *Vicia* specimens.

Species/Subspecies Name	Sample ID	GenBank Accession Number	Bit Score	*E*-Value	% Identity
*V. anatolica* Turill.	65	MZ338313	475	2E-137	99.61
*V. bithynica* L.	23	MZ338299	743	0	100%
*V. faba* L.	37	MZ338302	763	0	100%
*V. faba* L.	61	MZ338311	763	0	100%
*V. grandiflora* Scop.	55	MZ338309	621	0	99.71%
*V. grandiflora* Scop.	57	MZ338310	621	0	99.71%
*V. grandiflora* Scop.	71	MZ338318	580	2E-168	99.41%
*V. grandiflora* Scop.	70	MZ338317	627	0	100%
*V. lathyroides* L.	36	MZ338301	737	0	99.75%
*V. lathyroides* L.	38	MZ338303	737	0	99.75%
*V. lathyroides* L.	46	MZ338306	752	0	99.76%
*V. lathyroides* L.	49	MZ338308	758	0	100%
*V. lutea* L.	2	MZ338297	743	0	100%
*V. lutea* L.	45	MZ338305	743	0	100%
*V. lutea* L.	48	MZ338307	743	0	100%
*V. pannonica* Crantz	64	MZ338312	739	0	99.75%
*V. pannonica* Crantz	76	MZ338319	743	0	100%
*V. pannonica* Crantz	15	MZ338298	743	0	100%
*V. pannonica* Crantz	77	MZ338320	743	0	100%
*V. sativa* L.	66	MZ338314	763	0	99.52%
*V. sativa* L.	67	MZ338315	780	0	100%
*V. sativa* L.	80	MZ338322	778	0	100%
*V. sativa* L.	69	MZ338316	771	0	100%
*V. sativa* L.	79	MZ338321	771	0	99.76%
*V. sativa* L.	82	MZ338323	774	0	100%
*V. sericocarpa* Fenzl	32	MZ338300	353	5E-93	98.03%
*V. sericocarpa* Fenzl	40	MZ338304	353	5E-93	98.03%

**Table 3:** *rpoC1* BLAST analysis of selected *Vicia* specimens.

Species/Subspecies Name	Sample ID	GenBank Accession Number	Bit Score	*E*-Value	% Identity
*V. anatolica* Turill.	5	MZ285764	ǂ	ǂ	ǂ
*V. anatolica* Turill.	65	MZ285781	ǂ	ǂ	ǂ
*V. dionysiensis*	12	MZ285766	ǂ	ǂ	ǂ
*V. dionysiensis*	16	MZ285767	ǂ	ǂ	ǂ
*V. dionysiensis*	52	MZ285775	ǂ	ǂ	ǂ
*V. faba* L.	37	MZ285772	857	0	100%
*V. faba* L.	61	MZ285780	857	0	100%
*V. faba* L.	22	MZ285769	857	0	100%
*V. grandiflora* Scop.	55	MZ285777	ǂ	ǂ	ǂ
*V. grandiflora* Scop.	57	MZ285778	ǂ	ǂ	ǂ
*V. grandiflora* Scop.	71	MZ285786	ǂ	ǂ	ǂ
*V. grandiflora* Scop.	70	MZ285785	ǂ	ǂ	ǂ
*V. lathyroides* L,	38	MZ285773	ǂ	ǂ	ǂ
*V. michauxii* Spreng.	11	MZ285765	ǂ	ǂ	ǂ
*V. michauxii* Spreng.	19	MZ285768	ǂ	ǂ	ǂ
*V. pannonica* Crantz	85	MZ285790	ǂ	ǂ	ǂ
*V. peregrina* L.	35	MZ285771	ǂ	ǂ	ǂ
*V. sativa* L.	66	MZ285782	857	0	100%
*V. sativa* L.	59	MZ285779	857	0	100%
*V. sativa* L.	67	MZ285783	857	0	100%
*V. sativa* L.	80	MZ285788	857	0	100%
*V. sativa* L.	69	MZ285784	857	0	100%
*V. sativa* L.	79	MZ285787	857	0	100%
*V. sativa* L.	82	MZ285789	857	0	100%
*V. sericocarpa* Fenzl	32	MZ285770	ǂ	ǂ	ǂ
*V. sericocarpa* Fenzl	40	MZ285774	ǂ	ǂ	ǂ
*V. sericocarpa* Fenzl	53	MZ285776	ǂ	ǂ	ǂ

ǂ Species sequences missing from the GenBank database.

**Table 4:** *Vicia* species used in this study.

Species/Subspecies Name	Sample ID	Source
*V. aintabensis* Boiss.	3	Italy
*V. aintabensis* Boiss	13	France
*V. aintabensis* Boiss	20	Syria
*V. anatolica* Turill.	5	Turkmenistan
*V. anatolica* Turill.	50	Turkey
*V. anatolica* Turill.	54	Turkey
*V. anatolica* Turill.	65	Australia
*V. bithynica* L.	23	Syria
*V. bithynica* L.	43	Malta
*V. bithynica* L.	68	Azerbaijan
*V. bithynica* L.	72	Syria
*V. dionysiensis*	12	Syria
*V. dionysiensis*	16	Syria
*V. dionysiensis*	52	Syria
*V. ervilia* L.	8	Syria
*V. ervilia* L.	17	Syria
*V. ervilia* L.	34	Syria
*V. ervilia* L.	44	Syria
*V. ervilia* L.	78	Syria
*V. faba* L.	37	Syria
*V. faba* L.	61	Syria
*V. faba* L.	22	Syria
*V. grandiflora* Scop.	55	Armenia
*V. grandiflora* Scop.	57	Armenia
*V. grandiflora* Scop.	71	Sweden
*V. grandiflora* Scop.	70	Turkey
*V. hyaeniscyamus* Mouterde	41	Syria
*V. hyaeniscyamus* Mouterde	86	Syria
*V. hybrida* L.	25	Iraq
*V. hybrida* L.	42	Jordan
*V. hybrida* L.	60	Italy
*V. hybrida* L.	62	Morocco
*V. lathyroides* L.	36	Turkey
*V. lathyroides* L.	38	Turkey
*V. lathyroides* L.	46	Armenia
*V. lathyroides* L.	49	Algeria
*V. lutea* L.	2	Tunisia
*V. lutea* L.	45	Azerbaijan
*V. lutea* L.	26	Algeria
*V. lutea* L.	48	Turkey
*V. michauxii* Spreng.	11	Turkey
*V. michauxii* Spreng.	19	Tajikistan
*V. monantha* Retz.	21	Syria
*V. narbonensis* L.	1	Lebanon
*V. narbonensis* L.	4	Turkey
*V. narbonensis* L.	30	Syria
*V. narbonensis* L.	14	Syria
*V. narbonensis* L.	47	Jordan
*V. noeana* Boiss.	7	Syria
*V. noeana* Boiss.	9	Turkey
*V. noeana* Boiss.	29	Syria
*V. pannonica Crantz*	85	Uzbekistan
*V. pannonica Crantz*	64	Australia
*V. pannonica Crantz*	76	Italy
*V. pannonica Crantz*	15	Italy
*V. pannonica Crantz*	77	Turkey
*V. peregrina* L.	35	Iraq
*V. peregrina* L.	51	Syria
*V. peregrina* L.	63	Turkey
*V. peregrina* L.	73	Armenia
*V. sativa* L.	66	Egypt
*V. sativa* L.	59	Syria
*V. sativa* L.	67	Turkey
*V. sativa* L.	80	Market
*V. sativa* L.	69	Lithuania
*V. sativa* L.	79	Italy
*V. sativa* L.	82	Romania
*V. sericocarpa* Fenzl	32	Turkey
*V. sericocarpa* Fenzl	40	Iraq
*V. sericocarpa* Fenzl	53	Turkey
*V. villosa* Roth	24	Turkey

## Data Availability

The generated DNA barcoding data are available through GenBank (https://www.ncbi.nlm.nih.gov/genbank/) with accession numbers MZ334891-MZ334961 for *trnL*, accession numbers MZ338297-MZ338323 for ITS2, and accession numbers MZ285764-MZ285790 for *rpoC1*.

## Supplementary Materials

The following are available online at https://www.mdpi.com/article/10.3390/plants11070947/s1. Table S1: Multiple sequence alignment statistics, Table S2: Primers used in this study, Table S3: Best Maximum Likelihood model fit for the reconstruction of the *trnL*, ITS2, and *rpoC1* trees.
