# Supplementary material for: DNA-Based Identification of Eurasian Vicia Species Using Chloroplast and Nuclear DNA Barcodes
Source: Plants (Basel). 2022 Mar 31;11(7):947. doi: 10.3390/plants11070947 (PMC9003045; doi:10.3390/plants11070947)
Supplement: Supplementary file 1 [file plants-11-00947-s001.zip › plants-1609686-supplementary.pdf]

Table S1. Multiple sequence alignment statistics.

Barcoding region	*trnL*	ITS2	*rpoC1*
Pi	130	37	25
Co	180	203	405
Va	207	200	37
Si	77	162	12
Total	492	436	442

Pi: Parsimony-informative sites, Co: Conserved sites, Va: Variable sites, Si: Singleton sites.

Table S2. Primers used in this study.

Region	Primer	Reference
trnL-F	5'-CGAAATCGGTAGACGCTACG-3'	Taberlet et al., 2007
trnL-R	5'-GGGGATAGAGGGACTTGAAC-3'	Taberlet et al., 2007
rpoC1-F	5'-GGCAAAGAGGGAAGATTTCG-3'	Hollingsworth et al., 2009
rpoC1-R	5'-CCATAAGCATATCTTGAGTTGG-3'	Hollingsworth et al., 2009
ITS2-F	5'-ATGCGATACTTGGTGTGAAT-3'	Chen et al., 2010
ITS2-R	5'-GACGCTTCTCCAGACTACAAT-3'	Chen et al., 2010

Table S3. Best Maximum Likelihood model fit for the reconstruction of the *trnL*, ITS2, and *rpoC1* trees. Fitness assessment was performed for each alignment testing 24 nucleotide substitution models. The models with the lowest BIC score, which describe the substitution pattern the best, were selected for phylogenetic tree reconstructions.

Barcoding region	Model	BIC	AICc	lnL	Gamma	R
trnL	T92+G	3668.376	2317.342	-978.518	0.693038	0.746764
**ITS2**	K2+G	2854.923	2359.526	-1110.27	2.119952	1.279238
rpoC	JC+G	2415.15	1981.103	-932.292	0.05	0.5

BIC: Bayesian Information Criterion, AICc: Akaike Information Criterion, lnL: Maximum Likelihood value, R: transition/transversion bias.
